# A Basis for Dental Nomenclature

**Published:** 1895-12

**Authors:** A. H. Thompson

**Affiliations:** Topeka, Kan.


### A Basis for Dental Nomenclature.

BY A. H. THOMPSON, D.D.S., TOPEKA, KAN.

Abstract of paper read at American Dental Association, August, 1895.

It is generally conceded that our nomenclature is greatly in need of correction and codification, and to that end the first thing to be considered is a starting-point. To all students of the subject it would seem that this is furnished already to our hand by Dr. G. V. Black, in his admirable study and *resume* of the subject given before the World's Columbian Dental Congress and published in the Transactions (vol. ii. p. 825). This great paper is a landmark in the history of dental nomenclature, and may well serve as a starting-point for a new departure. Without wasting time in the discussion of the desirability of having an established nomenclature, on which we are all agreed, we might as well proceed at once to formulate a code arbitrarily. This can best be done by the American Dental Association, which is the national representative body of the profession in this country, the exponent of the opinion and policy of the profession, and as having authority in deciding questions of general interest to the profession. A decision emanating from this body would be held in respect by the profession, and the code of terms of nomenclature would be final and would gradually come into acceptance and use.

A list of the terms in general use should be submitted this year, to be known as "The Code of 1895," and lie over for one year to allow time for discussion and criticism by the profession at large, and then be adopted, with whatever corrections and amendments may seem desirable, at the ensuing annual meeting. Lists of terms in all departments can be added to this code from year to year as may seem desirable, by being proposed and submitted, laid over for one year for criticism and discussion, and then adopted. Newer terms in regard to some disputed points will provoke discussion, but they must be decided, and decided finally. A year's discussion will probably result in satisfactory terms for the points in dispute.

The writer is disposed to think that terms can be borrowed from the naturalists in naming the parts of teeth, that will dispense with many cumbrous combinations now in use in our literature. Some are offered in the list, and others will be included in the list of names for comparative dental anatomy.

A systematic dental nomenclature should be founded on the zoological system of names of the teeth of mammals employed by naturalists as a basis; on this should be placed a professional nomenclature, like Dr. Black's for the use of dentists, and on this a still more minute mapping system for the localizing of cavities, something like that prepared by Dr. Kulp. The three systems are indispensable. First, the gross description of teeth; second, the detailed descriptions; and third, the minute localization of areas on the surface of the teeth. Some arbitrary system of localizing cavities, like Dr. Kulp's, is desirable to avoid the cumbrous combinations of compound words which would otherwise be necessary. Dr. Kulp's plan of mapping the crown is the best of the kind, perhaps, yet offered. This is a matter that must be considered wisely before the final adoption of any system of minute description, such as is required in locating cavities.

DISCUSSION.

Dr. G. V. Black suggested that we formulate words and rules so far as we can, and then enforce the teaching of so much as has been determined in the schools. Then by keeping up the work of study and adoption as fast as definite conclusions shall have been reached, we shall have in a few years a nomenclature of which we may be proud. * * *

This association cannot alone establish a nomenclature, but it is the starting point through which it should be controlled.

The bast way for us, as has been done in other sciences, is to find some one with sufficient capacity, qualifications and the taste for the work, with a knowledge of the various languages, to take it up and be the leader. It requires not only one who has the necessary educational qualifications, but one also who has the inclination to work continuously.

Dr. T. C. Stellwagen suggested for students' use a small book in which is written the terms used, with their meanings. They are thus taught to use the words exactly as they are intended to be used, and so keep their edges keen. Only two hundred to three hundred words suffice for the ordinary uses of life among the uneducated.

Dr. T. E. Weeks said that the question just replied to emphasized the fact that in our pronunciation of terms, as well as in their proper use, there is still room for improvement. If the movement that has been set on foot is carefully and persistently followed out, great good will result. He hoped the committee would be continued, and that the association may adopt the work of an honest and earnest committee.

Dr. W. C. Barrett wished to call attention to certain solecisms and barbarisms which are very offensive, as "wisdom-tooth" for the third permanent molar, and "fangs" for roots of teeth. There is where, in his opinion, the work of reforming the nomenclature of dentistry should begin; these exceedingly gross violations of propriety and accuracy should first engage our attention.

Dr. A. H. Thompson: This matter of expunging objectionable and meaningless terms is a work of which the committee recognizes the importance, and they will do it as fast as they can; but they cannot do it all at once. Instances might be multiplied, almost without limit, of variations in the use of pronunciation of words. Thus of "alloy," two pronunciations are in use; of "cement," the same. We must have a distinct basis of understanding of all these matters, and the committee hope in time to have all the errors corrected.

Dr. John S. Marshall moved that the report be accepted and the committee continued, and that the list of words upon which they agree be submitted to the association for criticism and suggestion before final action is taken upon them. So ordered.

Dr. Guilford: Steele compared words to tools. It was an excellent comparison. Words are tools. Driving nails with a hammer, with a monkey-wrench, and with a rude piece of iron, this is analogous to what the dental profession has been doing in its use of words. This report was made to show what the committee had done. Every dental society ought to take up the work, and every man ought to constitute himself a committee of one to look the matter up for himself, and the next year we shall be prepared to act. Then, if the teachers in the colleges and the editors of the journals will use the terms agreed upon, a desirable change will soon be brought about.

Dr. Guilford then moved that Dr. Black be placed upon the committee in place of Dr. Stubblefield, who had been unable to act with it.

